# Image formation in diffusion MRI: A review of recent technical developments

**DOI:** 10.1002/jmri.25664

**Published:** 2017-02-14

**Authors:** Wenchuan Wu, Karla L. Miller

**Affiliations:** ^1^ FMRIB Centre, Nuffield Department of Clinical Neurosciences University of Oxford Oxford UK

**Keywords:** diffusion MRI, pulse sequence, image artifacts, accelerated imaging, high‐resolution imaging, navigation

## Abstract

Diffusion magnetic resonance imaging (MRI) is a standard imaging tool in clinical neurology, and is becoming increasingly important for neuroscience studies due to its ability to depict complex neuroanatomy (eg, white matter connectivity). Single‐shot echo‐planar imaging is currently the predominant formation method for diffusion MRI, but suffers from blurring, distortion, and low spatial resolution. A number of methods have been proposed to address these limitations and improve diffusion MRI acquisition. Here, the recent technical developments for image formation in diffusion MRI are reviewed. We discuss three areas of advance in diffusion MRI: improving image fidelity, accelerating acquisition, and increasing the signal‐to‐noise ratio.

**Level of Evidence:** 5

**Technical Efficacy:** Stage 1

J. MAGN. RESON. IMAGING 2017;46:646–662

Single‐shot echo planar imaging (SSH‐EPI) has been used as the standard image formation method for diffusion magnetic resonance imaging (MRI) on commercialized scanners for more than 20 years. This is mostly due to the fast acquisition speed of SSH‐EPI (100–200 msec per slice including diffusion preparation), which makes it fairly immune to subject motion and enables advanced diffusion protocols with a large number of diffusion directions and/or b‐values within reasonable scan times. However, SSH‐EPI is prone to several limitations, including image distortions due to B_0_ inhomogeneity at tissue/air interfaces and $T_{2}^{*}$ blurring, both of which place limitations on spatial resolution. High‐resolution diffusion MRI provides the ability to resolve fine‐scale structures, enabling detection of cortical anisotropy,^1^, ^2^ delineation of thin white matter tracts,^3^ and more accurate fiber tractography.^4^ Although parallel imaging has improved the data quality of SSH‐EPI, these problems still exist and become more pronounced at high field strength and high resolution.

Alternative acquisition schemes have been proposed to overcome the limitations of SSH‐EPI, including segmented‐EPI readout, non‐EPI trajectories, and reduced field of view (FOV). These methods have undergone rapid development in recent years, demonstrating significantly improved image quality compared to SSH‐EPI. Conventional 2D acquisition schemes suffer from long scan time and low signal‐to‐noise ratio (SNR) efficiency when acquiring high isotropic‐resolution diffusion MRI data with full brain coverage, which is increasingly needed in neuroscience studies. The recent development of simultaneous multislice techniques has dramatically changed this situation and diffusion MRI data can be acquired more rapidly and with higher SNR efficiency. Various 3D diffusion MRI acquisitions have also been developed, which have high SNR efficiency and can provide more accurate slice definitions than 2D acquisition. Several studies have reported high‐quality diffusion MRI data at ultrahigh field of 7T, which opens new possibilities for achieving higher spatial and angular resolution. Finally, developments for fast diffusion MRI using compressed sensing have been reported, which can further accelerate diffusion acquisition.

In this article we review recent developments in image formation methods for diffusion MRI and discuss how these are likely to be used in practice. We focus on three kinds of advances: improving image fidelity, accelerating acquisition, and increasing SNR. However, inevitably a method that impacts one of these metrics has consequences for the others, and we aim to describe these tradeoffs throughout the review. Some of the reviewed methods are fairly unique to diffusion imaging (eg, navigated correction of motion‐induced phase errors), while others have broader application (eg, simultaneous multislice imaging). For conciseness, we do not cover diffusion contrast mechanisms and use examples focusing on diffusion MRI of the brain.

## Improving Image Fidelity

**Figure 1:**
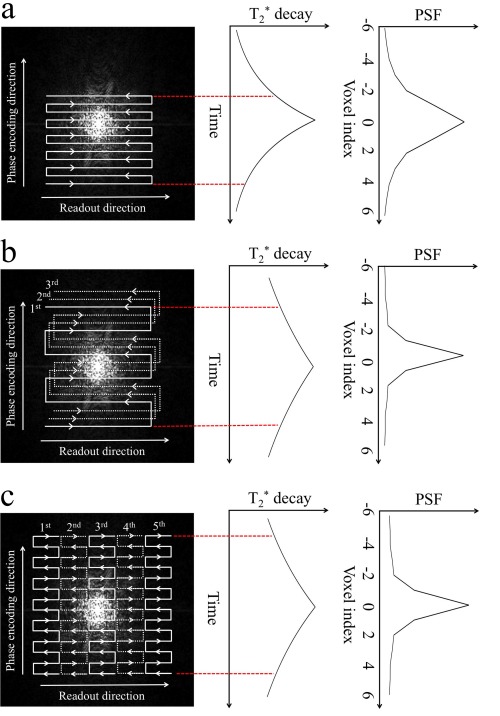
**(a)** Single‐shot EPI trajectory samples *k*‐space very rapidly (20–40 msec per image). However, tissue $T_{2}^{*}$ decay causes signal loss at outer *k*‐space, which corresponds to high spatial frequencies, leading to image blurring. This blurring is quantified by the point spread function (PSF), which describes the extent of blurring of signal from nearby voxels (ie, a wider PSF corresponds to more blurring). Partial Fourier acquisition is illustrated here, which can effectively reduce the echo time and hence increase SNR. **(b)** Conventional segmented EPI (three segments shown here) and **(c)** readout segmented EPI (five segments shown here) significantly reduces the effective echo spacing (eg, from about 0.8 msec in SSH‐EPI to about 0.25 msec in segmented EPI and 0.32 msec in readout segmented EPI; parallel imaging is not considered here), leading to sharper PSF shapes. Note that we are only considering $T_{2}^{'}$ decay, but that *T*_2_ decay will also be occurring. However, this effect does not in general change the PSF characteristics by much.

**Figure 2:**
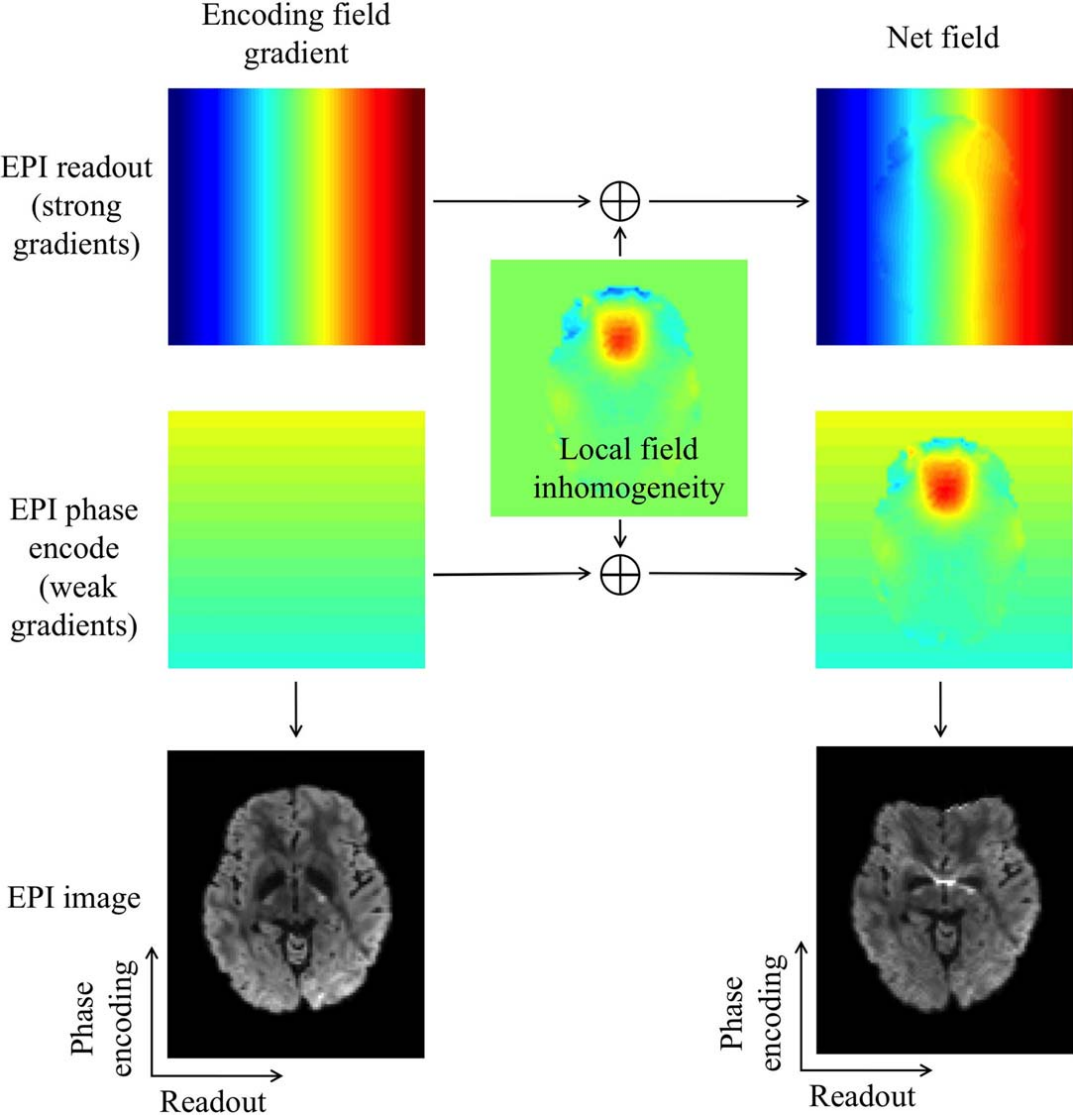
EPI distortions stem from inhomogeneity of the main magnetic field and are most pronounced at tissue/air and tissue/bone interfaces due to the large local field inhomogeneity caused by susceptibility variations. MR image formation assumes the linear field gradient used for spatial encoding is achieved exactly as planned. However, due to the main field inhomogeneity, the net field deviates from the desired linear change, leading to incorrect mapping of voxels. The result is image distortion (eg, compression in the frontal lobe, as shown in the figure). As the encoding field for EPI phase encode is relatively weak compared to local field changes, distortions are severe along this direction. Encoding field for EPI readout is much stronger than local field changes and the image distortion along this direction is negligible.

The two dominant image artifacts in SSH‐EPI are image blurring and distortion. The spatial resolution of SSH‐EPI is severely affected by the tissue $T_{2}^{*}$ decay (Fig. 1), which results in intense signal loss at the outermost edges of *k*‐space. Because outer *k*‐space corresponds to fine spatial detail (high spatial frequencies), this weighting introduces image blurring. Thus, a short readout window is important for reducing EPI blurring. Image distortion in SSH‐EPI mainly happens in regions with strong local magnetic field inhomogeneity (eg, tissue/air boundaries with fast susceptibility variation). This local field distortion can swamp the weak gradients used for phase encoding, resulting in misplaced signal that appears as image distortion (Fig. 2). The scale of distortion is determined by the speed of *k*‐space transversal along the phase‐encoding direction. Therefore, short echo spacing and undersampling, both of which enable faster traversal along the phase‐encoding direction, are desirable properties for improving SSH‐EPI. The application of parallel imaging^5^, ^6^ in SSH‐EPI has been very successful, although this method faces challenges from noise amplification, particularly for diffusion MRI. “Effective echo spacing” accounts for the reduced distortion in parallel imaging by dividing the acquired echo spacing by the acceleration factor, which gives the echo spacing that would have been required to achieve this level of distortion without acceleration (ie, any EPI scan with the same effective echo spacing will have the same distortion). Reduced FOV methods can reduce distortion without noise penalty, but is limited to small coverage.

Another source of distortion in diffusion MRI is the eddy currents induced within the conducting surfaces of the magnet due to fast gradient switching. Modern systems include gradient “pre‐emphasis” that can substantially reduce eddy currents, but these corrections struggle to compensate for strong diffusion‐encoding gradients with high slew‐rate. The twice‐refocused spin‐echo diffusion preparation^7^ can further reduce the effects of eddy currents, but at the cost of longer echo time. This is particularly problematic for high‐resolution scans at ultrahigh field, where this scheme requires ∼30 msec longer echo time (TE) than conventional spin‐echo diffusion preparation for 1 mm resolution scan, exacerbating the already problematic *T*_2_ signal loss. Another approach is to correct the distortion in postprocessing.^8^ More recently, correction of distortion based on nonparametric modeling of diffusion signal with respect to diffusion‐encoding direction has demonstrated excellent results.^9^

Partial Fourier acquisition is often used to reduce the long echo time of SSH‐EPI, which is another challenge for diffusion MRI due to greater *T*_2_ signal decay (lower SNR). This is particularly problematic for high‐resolution scans with longer echo time and smaller voxel size. Although partial Fourier reduces echo time, it increases the sensitivity to subject bulk motion during diffusion encoding,^10^ which induces echo shifting in *k*‐space.^11^ For strong motion, the echo is shifted toward the edge of *k*‐space (higher spatial frequency), violating the fundamental assumptions underlying partial Fourier and causing the reconstruction to fail. This leads to image intensity oscillations and signal loss.^12^ To alleviate this problem, adaptive partial Fourier reconstruction algorithms have been proposed.^12^, ^13^

Other common EPI artifacts that may appear in diffusion MRI images include Nyquist ghosting caused by hardware‐related odd–even echo misalignment and fat shifting along the phase‐encoding direction. Nyquist ghosting is typically corrected by measuring the *k*‐space shift between odd and even echoes using a reference scan and subsequently realigning the *k*‐space data. Reference‐less methods using image‐entropy as a selection metric have also demonstrated robust correction of Nyquist ghosting.^14^ Fat shifting occurs due to the difference of resonance frequencies between water and fat and the low bandwidth in the phase‐encoding direction. For example, because fat differs from water by 440 Hz at 3T, fat signal will be shifted by ∼1/3 FOV for an EPI acquisition with echo spacing of 0.8 msec = (1250 Hz)^−1^. To eliminate fat‐shifting artifacts, fat suppression is commonly implemented in EPI acquisition. Most fat suppression methods utilize special excitation schemes, including water‐only spectral spatial excitation, fat saturation (and spoiling), and inversion‐recovery preparation.^15^

**Figure 3:**
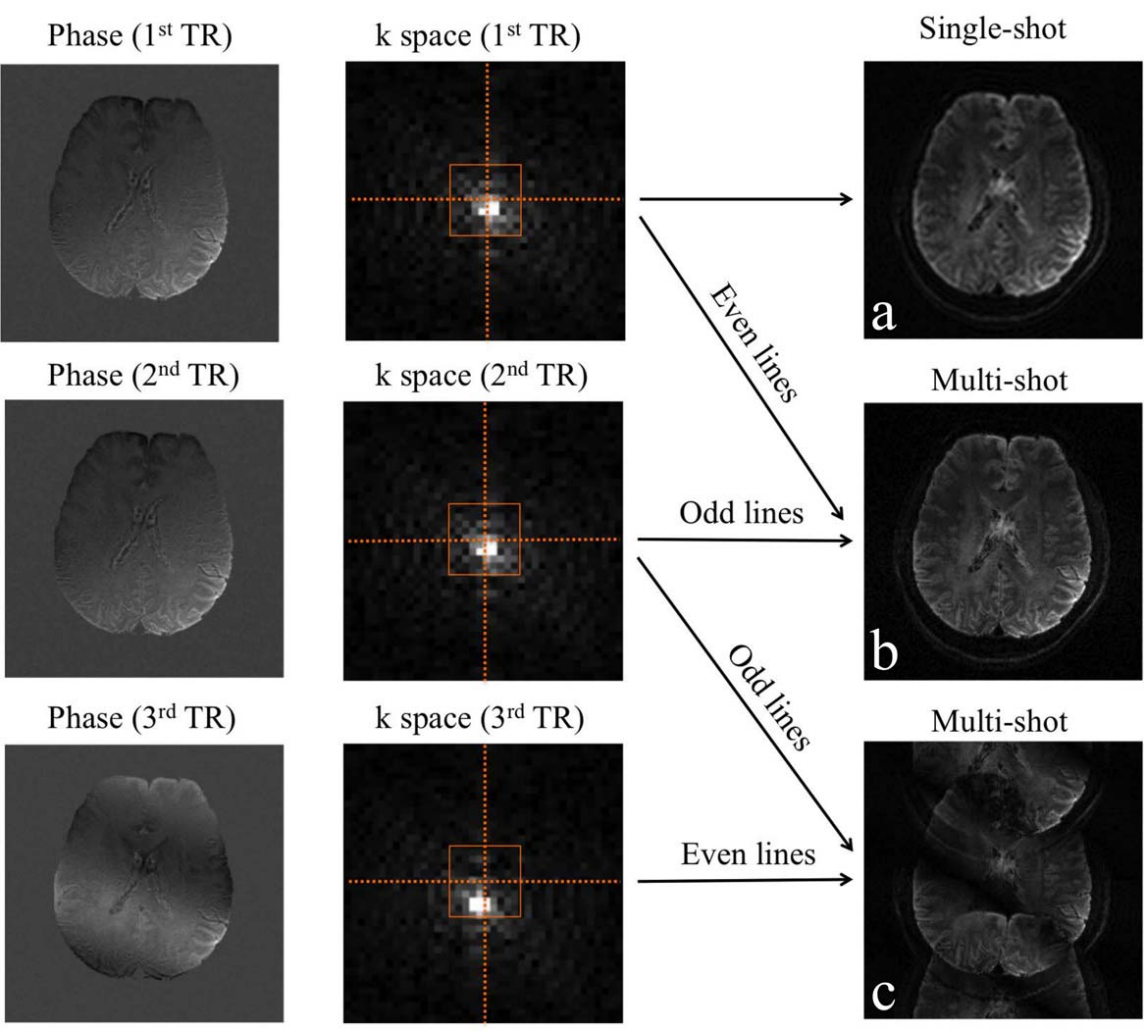
Subject motion during diffusion encoding can introduce substantial spatially varying phase to the data. For single‐shot EPI, this is not a problem, as only the magnitude is used and the phase is discarded **(a)**. For multishot acquisition, however, this phase inconsistency must be properly handled. In this illustration, the subject is assumed to be steady during the first two TRs (no phase changes) and have a head rotation during the third TR, which introduces a linear phase offset (corresponding to a shift in *k*‐space). Multishot acquisition using even lines from the 1^st^ TR and odd lines from the 2^nd^ TR provides an artifact‐free image **(b)**. Multishot acquisition using odd/even lines from the 2^nd^ and the 3^rd^ TRs in a similar manner suffers from substantial image corruption due to the shot‐to‐shot phase inconsistency **(c)**. One solution to this problem is navigation, which measures the motion‐induced phase errors during each shot and correct them before combining all segments.

A number of multishot acquisition techniques have also been proposed, for which subject motion must be carefully handled, typically using a *k*‐space “navigator.” Navigators provide an unaliased, low‐resolution image corresponding to a limited central *k*‐space region that is used to predict effects from subject motion. Navigators are usually acquired immediately before/after the imaging data or extracted from the imaging data directly (“self‐navigation”). Diffusion signals are intrinsically sensitive to subject motion because diffusion preparation gradients encode tiny (molecular) motions in signal phase. Even small subject motions (eg, cardiac pulsation, respiration) during diffusion preparation can lead to substantial spatially varying phase that is unrelated to diffusion. SSH‐EPI is immune to these phase errors because it captures the entire image in one shot, such that the phase of the signal can be discarded, while diffusive motion is reflected in the signal magnitude. By comparison, multishot image acquisitions must retain phase information in order to accurately combine across the different *k*‐space segments acquired in each shot. If motion‐induced phase is not corrected before combining multishot segments, images become severely corrupted (Fig. 3). To correct motion‐induced phase errors, it is common to acquire additional *k*‐space measurements that can be used as a low‐resolution navigator, using the phase of the navigator image to rectify the phase inconsistency between segments.

### Readout‐Segmented EPI

Readout‐segmented EPI (rs‐EPI) uses a series of EPI acquisitions to cover *k*‐space in a mosaic pattern,^16^ as depicted in Fig. 1c. By acquiring concatenated *k*‐space segments along the readout direction, rs‐EPI can achieve much shorter echo spacing (eg, echo spacing could be shortened from about 0.8 msec in SSH‐EPI to about 0.3 msec in rs‐EPI with 7 readout segments for diffusion scan at 1.2 mm isotropic‐resolution^17^), and hence considerably reduce geometric distortion and $T_{2}^{*}$ blurring.^18^, ^19^, ^20^, ^21^ The effective echo spacing can be further reduced via the combination with parallel imaging.^5^, ^6^ The resulting TE of rs‐EPI is also shorter than SSH‐EPI (eg, for 2 mm^2^ isotropic‐resolution scan and b = 1000s/mm^2^ at 3T, TE could be reduced from about 87 msec in SSH‐EPI to about 73 msec in rs‐EPI,^17^ which is predicted to result in an ∼25% SNR improvement). The improvements offered by the reduced echo spacing (reduced distortion, blurring, and TE) should be particularly beneficial at ultrahigh field, where tissue *T*_2_ and $T_{2}^{*}$ are very short and field inhomogeneity is worse.^22^

As a multishot diffusion acquisition method, rs‐EPI requires navigation to remove motion‐induced phase errors. rs‐EPI acquires a continuous *k*‐space segment, enabling fast and robust correction using the nonlinear phase correction method.^19^, ^20^ By comparison, in conventional segmented EPI (see “Segmented EPI” section, below), the image reconstructed from each segment suffers from aliasing, which is difficult to correct robustly using the nonlinear phase correction. Iterative algorithms^23^ can address this problem, but at the cost of long computational time.

The nonlinear phase correction only works if the motion‐induced phase errors can be accurately extracted from the navigator. In case of severe subject motion during diffusion preparation, the navigator fails to correct the phase errors when the center of *k*‐space shifts out of the navigator acquisition window, making the motion information obtained from the navigator inaccurate. These corrupted data could severely degrade the image quality, and they cannot be simply removed as in Propeller^24^ (see “Propeller” section, below) because there is no redundancy between readout segments. Instead, severely corrupted segments can be detected using the navigator and replaced by reacquired segments at the same *k*‐space location.^20^

As with other multishot methods, the primary challenge of rs‐EPI is the longer scan times required to form each image volume. Many of the approaches to reducing scan times discussed in the section on acceleration of diffusion MRI are compatible with rs‐EPI, and both partial Fourier^25^ and simultaneously multislice^17^ have been proposed to reduce scan times. rs‐EPI has also been demonstrated at 7T^22^ and in conjunction with 3D multislab acquisitions.^26^

Superior data quality using rs‐EPI has been demonstrated compared to SSH‐EPI, especially in regions with strong susceptibility variation, such as the temporal lobes and brainstem.^19^, ^20^ Several clinical studies have investigated the performance of rs‐EPI for diagnostics, demonstrating high data fidelity and improved conspicuity of pathology, for applications including: breast cancer,^27^, ^28^, ^29^ liver tumors,^30^ pelvic,^31^, ^32^ and renal^33^ diseases, and pediatric neuropathology.^34^

### Segmented EPI

Segmented EPI samples *k*‐space over multiple EPI trajectories with broadly spaced lines that interleave to cover the full *k*‐space, as depicted in Fig 1b. In early implementations, a 2D navigator was used to correct the motion‐induced phase errors.^35^, ^36^

Recently, the multiplexed sensitivity encoding (MUSE) method^37^ has been proposed to correct the motion‐induced phase errors in segmented EPI without acquiring 2D navigators. MUSE reconstruction consists of three components: first, a phase navigator is calculated for each segment using parallel imaging to fill in the missing samples in central *k*‐space; second, each segment is phase‐error‐corrected using this navigator; third, all segments are combined to form the final image (in practice, the second and third parts are calculated simultaneously). Several refinements of MUSE were proposed to correct the rigid motion between different segments (Fig. 5)^38^, ^39^ and prospectively detect and reject severely motion‐corrupted interleaves.^40^ Extensions of MUSE with robust partial Fourier reconstruction, simultaneous multislice,^13^ and 3D multislab acquisition have also been proposed.^41^

A disadvantage of the MUSE method is the limitation on the number of interleaves, since this equates to the acceleration factor in the first reconstruction step, which is constrained by the receiver coil design. A more recent method formulated the reconstruction as a low‐rank matrix completion process without explicitly estimating the motion‐induced phase errors.^42^ These navigator‐free methods improve the efficiency of segmented EPI acquisition (navigator acquisition takes 30–40 msec for each excitation) and reduce the specific absorption rate (SAR) (by ∼30% due to the removal of the refocusing pulse for the navigator echo), which may be critical at ultrahigh field.

### Propeller

**Figure 4:**
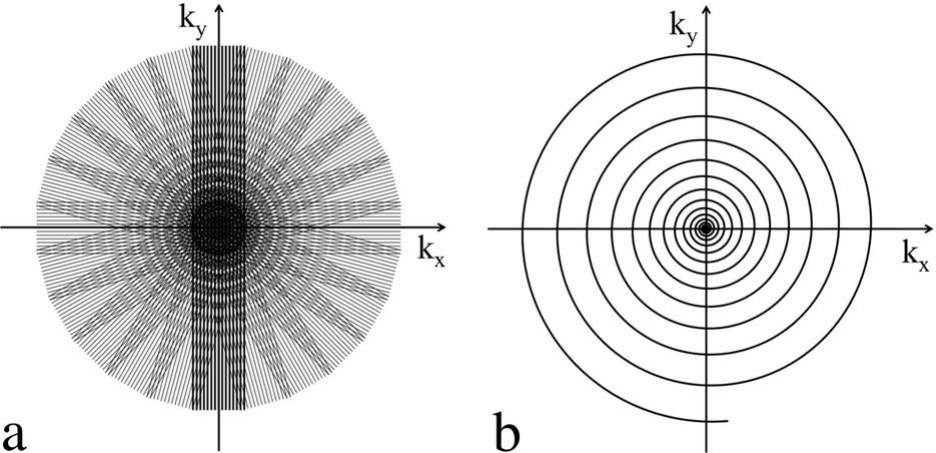
Two non‐Cartesian diffusion acquisition methods: **(a)** PROPELLER; **(b)** variable density spiral.

One approach to reducing image distortion and $T_{2}^{*}$ blurring uses a class of sequences known as fast spin echo (FSE, sometimes called TSE or RARE),^43^ which uses a series of refocusing pulses to create a train of spin echoes. This method can reduce artifacts by acquiring every *k*‐space line at the center of a spin echo, thus avoiding the phase accumulation that leads to distortion and blurring in EPI. The most widely used implementation of FSE in diffusion MRI is PROPELLER (Periodically Rotated Overlapping ParallEL Lines with Enhanced Reconstruction), which acquires a strip (“blade”) covering the center of the *k*‐space in each train of refocusing pulses.^24^, ^44^ Multiple rotated blades are acquired to fully sample a circular *k*‐space region over multiple shots, as shown in Fig 4a. A key strength of PROPELLER is that it is self‐navigating: all blades cover the *k*‐space center, which can be used to estimate the motion‐induced phase errors for each shot.

**Figure 5:**
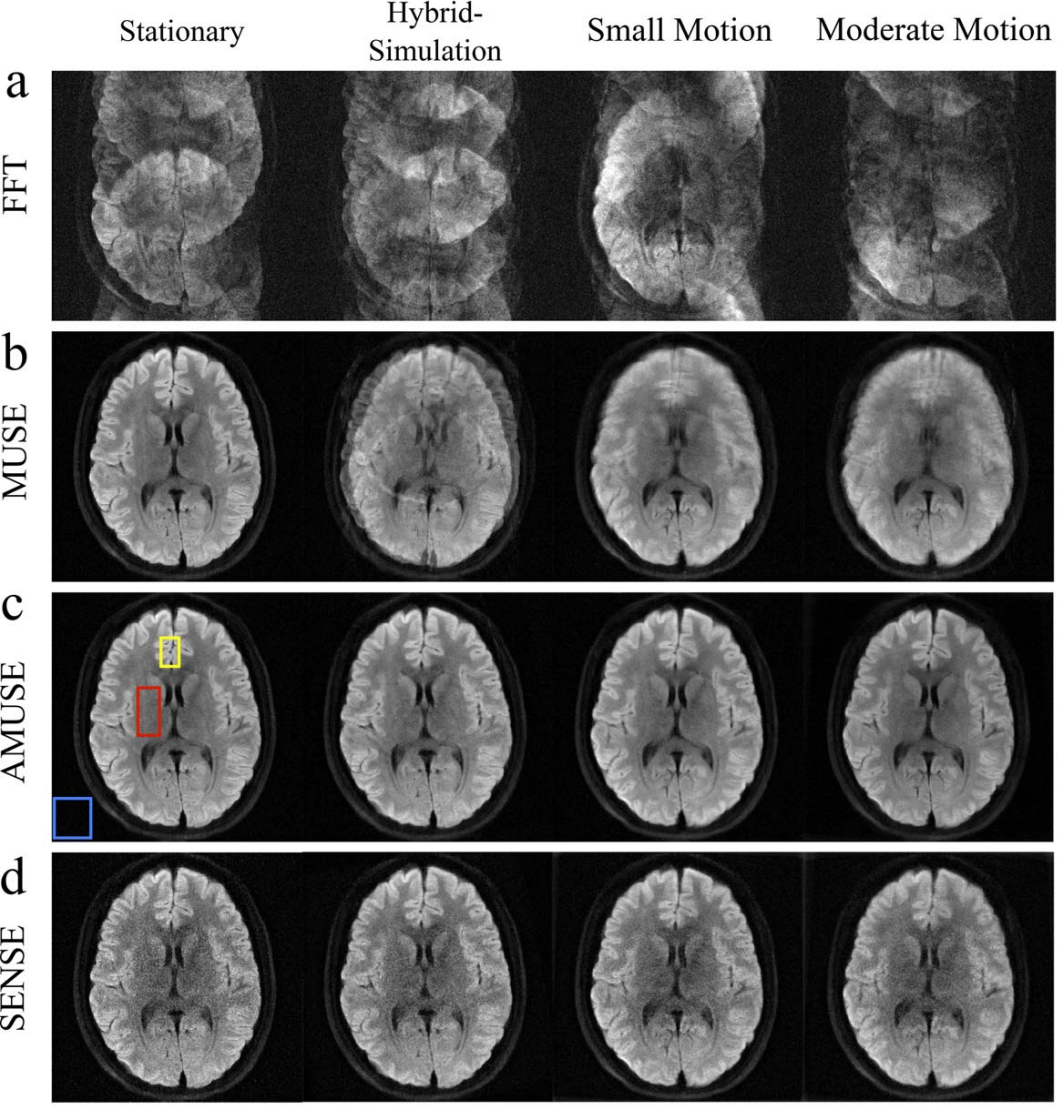
Reconstruction of multishot diffusion MRI data using direct fast Fourier transform (FFT), multiplexed sensitivity encoding (MUSE), augmented MUSE (AMUSE), and sensitivity encoding (SENSE)‐based motion correction. The AMUSE method simultaneously corrects motion‐induced phase errors and macroscopic motion. Four types of subject motion are evaluated, including stationary, hybrid‐simulation (combining data from two scans, in which subject head is stationary during acquisition but rotates about 15° between scans), small motion (about ± 5° rotation every 10–15 sec) and moderate motion (about ± 10° rotation every 10–15 sec). FFT and MUSE are corrupted by subject motion, whereas both AMUSE and SENSE reduce the motion artifacts. AMUSE further provides higher SNR. Figure reproduced with permission from Ref. 38.

**Figure 6:**
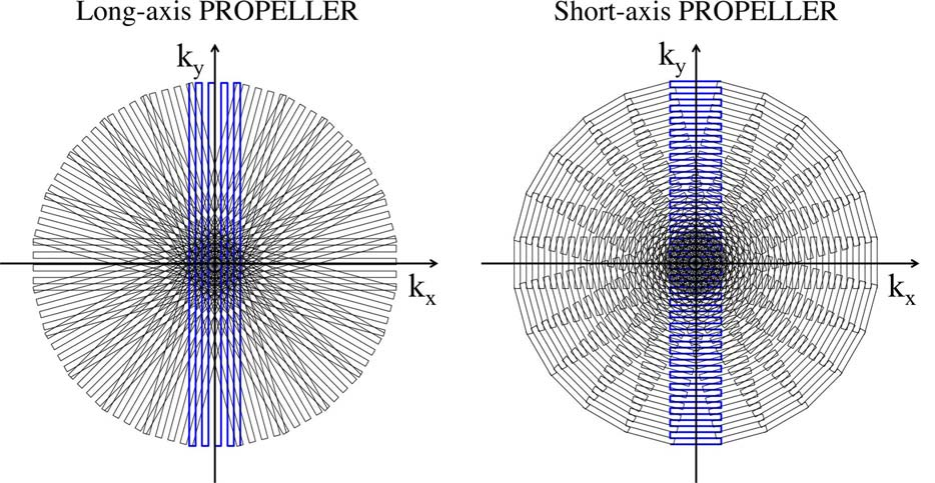
Two variants of PROPELLER‐EPI: long‐axis (LAP) PROPELLER and short‐axis (SAP) PROPELLER. The readout directions for LAP and SAP are along the long‐ and the short‐axis of the strip, respectively. The k_y_ transverse speed in SAP acquisition is faster than that in LAP, resulting in fewer blurring artifacts.

Several key challenges for PROPELLER have been identified and addressed, including motion sensitivity (due to the conditions for formation of spin‐echo trains), RF deposition (due to the large number of refocusing pulses in the FSE readout), and imaging speed (due to the multishot acquisition). Signal formation requires stable signal phase over the course of the spin‐echo train,^45^ which is disrupted by motion, resulting in signal decay and oscillates. Alternating the phase of the refocusing pulses between x and y axes has been demonstrated to stabilize the signal.^24^, ^46^, ^47^ To accelerate the acquisition and reduce RF deposition, Turboprop^48^ collects multiple gradient echoes between refocusing pulse pairs. Turboprop is in effect a gradient‐ and spin‐echo (GRASE^49^) sequence, providing a tradeoff between image distortion and speed. Going further in this direction, PROPELLER‐EPI^50^ acquires each blade using a single EPI readout, combining the self‐navigation of PROPELLER and the rapid acquisition and low‐SAR properties of EPI. PROPELLER‐EPI has greater blurring and distortion than the original FSE technique, which can be mitigated with parallel imaging.^51^ Alternatively, short‐axis PROPELLER‐EPI^52^ places the EPI readout along the short axis of the blade (Fig. 6), leading to short echo spacing, and therefore reduced blurring artifacts.

### Spiral

The most commonly considered alternative to Cartesian sampling techniques like EPI are spiral trajectories, in which *k*‐space is traced in a radiating pattern rather than a line‐by‐line scan. Spiral imaging has the merit of intrinsic motion compensation through gradient‐moment‐nulling^53^ and efficient use of gradient power.^54^ Single‐shot spiral imaging acquires diffusion MRI data with similar efficiency as SSH‐EPI,^55^ but suffers from different artifacts due to off‐resonance and $T_{2}^{*}$ decay, both of which result in image blurring.

Similar to segmented EPI, spiral acquisitions can be acquired in multiple shots using interleaved acquisitions to achieve high spatial resolution and reduced image blurring. As with all multishot diffusion imaging methods, motion‐induced phase errors need to be corrected before combining interleaved spiral acquisitions. Variable density spirals (VDS) can be self‐navigating by sampling central *k*‐space densely with each interleave (Fig. 4b).^56^ Alternatively, constant density spirals can be used with a similar strategy as MUSE to extract motion navigators using a parallel imaging reconstruction.^57^ Spirals are also pseudo‐incoherent with respect to undersampling artifacts, which makes it a preferable sampling method for compressed sensing reconstruction,^58^ as discussed below. Spirals have also been extended to 3D acquisitions using thin slabs.^59^ The major challenge for spiral acquisition is image blurring, which compromises the spatial resolution that can be achieved. Although deblurring methods can alleviate this problem to some extent, their performance still needs to be improved in the presence of strong susceptibility variations or severe B_0_ inhomogeneity (eg, ultrahigh field).

### Reduced FOV Methods

Similar to parallel imaging,^5^, ^6^ reduced field of view (rFOV) techniques reduce distortion and $T_{2}^{*}$ blurring by skipping phase‐encoding lines. In rFOV, aliasing is avoided by excluding signal from outside a limited volume within a region of extended tissue. Three main strategies for rFOV acquisition and their application in diffusion MRI are reviewed here: inner volume imaging, outer volume suppression, and multidimensional RF excitation.

Inner volume imaging (IVI) uses orthogonal orientations for excitation and refocusing pulses such that only the overlapping regions of the excited volume and refocused volume create signal, enabling a reduced imaging FOV.^60^ In its original form, IVI was limited to single‐slice imaging because the refocusing pulse saturates parallel slices. A refinement of the IVI method places the refocusing pulse at a shallower angle to the excitation,^61^ which enables multiple slice acquisition, but requires gaps between slices. Alternatively, one can apply another refocusing pulse after the readout,^62^ returning the spins from the non‐imaged slices to the positive longitudinal axis, which enables contiguous interleaved multislice acquisition.

**Figure 7:**
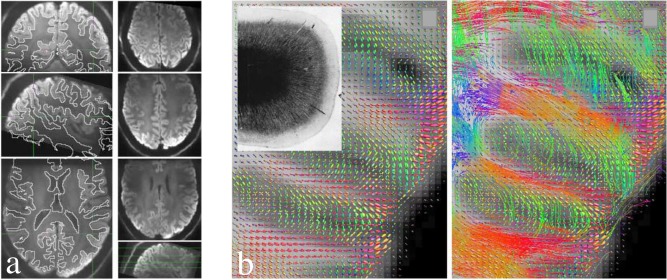
High‐resolution (0.8 mm isotropic) diffusion MRI data acquired using a combination of reduced FOV and parallel imaging method at 7T. **(a)** Left column: Trace‐weighted images overlaid with white/gray matter boundaries obtained from an anatomical scan, demonstrating high geometric fidelity achieved by combining reduced FOV methods and parallel imaging. Right column: axial slices at different brain regions. **(b)** Fiber orientation density (left) and streamline tracking (right) based on the high‐resolution data, depicting white matter fiber tracts entering the cortex. Figure reproduced with permission from Ref. 66.

Outer volume suppression (OVS) suppresses signal from outside the imaging volume using spatially selective RF pulses followed by dephasing gradients.^63^ The OVS pulses are applied prior to the imaging acquisition, resulting in signal only from the nonsuppressed target region. OVS incurs increased SAR and longer scan time due to these suppression pulses, and is sensitive to RF transmit field inhomogeneity. Nevertheless, reduced FOV diffusion MRI with OVS has demonstrated superior structural details in spinal cord^63^ and pons^64^ compared to conventional SSH‐EPI. In combination with parallel imaging, OVS has been used to address the severe B_0_ inhomogeneity and short tissue *T*_2_ value at ultrahigh field (Fig. 7).^65^, ^66^, ^67^ OVS has also been combined with SMS for high‐resolution diffusion MRI.^68^, ^69^

The third approach to reduced FOV uses 2D spatially selective RF pulse for excitation and a conventional slice‐selective 180° pulse for refocusing.^70^ The most common multidimensional pulses, using echo‐planar gradients, result in a periodic excitation profile. This profile places limits on the orientation and number of slices.^71^ A refinement of this method demonstrated the ability to simultaneously refocus two slices,^72^ doubling the number of slices that can be acquired in each scan. An alternate approach has been proposed that has virtually unlimited slice coverage, but which requires separate fat saturation.^73^ The long pulse durations associated with multidimensional excitations can be reduced using parallel transmission.^74^ Multidimensional excitation for rFOV diffusion imaging has been compared to the standard SSH‐EPI method, demonstrating clinical feasibility and improved conspicuity for spinal cord^75^, ^76^ and breast imaging.^77^, ^78^, ^79^

## Accelerating Diffusion MRI Acquisitions

As noted above, SSH‐EPI is highly efficient during the signal readout period, providing all the spatial information for one slice in 20–40 msec. However, regardless of readout, diffusion MRI sequences are generally inefficient, with ≤50% of the sequence time dedicated to signal acquisition due to the need for a long diffusion preparation. In conventional 2D SSH‐EPI sequences, this inefficiency is compounded by the fact that each slice is encoded independently in series, such that the acquisition time per volume scales with the number of imaging slices. Assuming fixed coverage, increased spatial resolution requires more slices, further inflating volume scan time. This inefficiency results in a difficult tradeoff, particularly when a large number of diffusion directions are desired to improve the accuracy of angular information (eg, for diffusion tractography). When total scan time is limited, there is a fundamental tradeoff between spatial coverage, spatial resolution, and angular resolution (density of sampling in the diffusion‐encoding [directional] domain).

Several techniques have been introduced in the past few years that have the potential to dramatically reduce this tradeoff. Simultaneous multislice (SMS) techniques have provided the ability to acquire multiple diffusion‐encoded slices simultaneously, increasing the scan efficiency (as reflected in the number of slices acquired per unit time). Compressed sensing has shown the possibility to reconstruct MRI image from highly undersampled data,^58^ which can benefit diffusion scans with a large number of diffusion directions and/or multishot *k*‐space acquisition.

### Simultaneous Multislice Imaging

The idea of exciting multiple slices simultaneously was proposed more than 20 years ago.^80^, ^81^ However, the first SMS techniques required multiple excitations to separate slices and did not reduce scan time. A key advance was made when it was realized that multichannel coil arrays enabled slice separation from a single acquisition through a parallel imaging formulation, thereby accelerating volume acquisition.^82^ This approach was subsequently extended to SSH‐EPI^83^ and demonstrated enabling high spatial‐angular resolution diffusion MRI.^84^, ^85^

**Figure 8:**
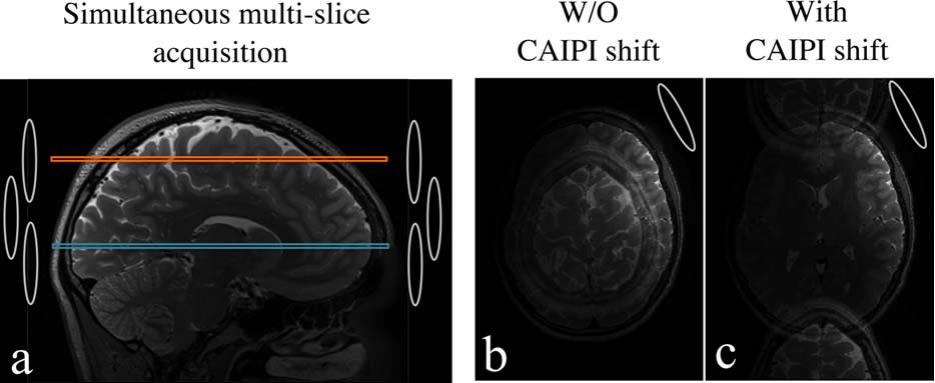
Simultaneous multislice imaging utilizes multiple‐channel coil arrays to separate multiple slices excited simultaneously (shown in orange and blue in **(a)**). The collapsed slices can be separated using parallel imaging, given there are sufficient variations in the coil sensitivities. However, in the case of high slice acceleration, the distances between the aliased voxels become small, making it difficult to resolve the aliasing due to the lack of coil sensitivity variations **(b)**. The result is residual artifacts and amplified noise (g‐factor penalty) in the reconstructed images. With CAIPI scheme (eg, blipped‐CAIPI in SSH‐EPI), the distances between slices are increased by an apparent shifting along the phase‐encoding direction **(c)**, which can significantly improve the reconstruction.

A major challenge faced by SMS is noise amplification when gaps between slices are small. Coil profiles in general vary slowly across space, meaning that closely separated slices tend to have similar profiles (ie, the high‐signal region from one slice overlaps with the high‐signal region from another slice) (Fig 8b). This problem is the SMS manifestation of the “g‐factor” (the noise amplification for a given image voxel, which reflects coil configuration, acquisition protocol, and reconstruction algorithm) from conventional parallel imaging^5^ and is particularly challenging for diffusion MRI due to its low intrinsic SNR. The “blipped‐CAIPI” (controlled aliasing in parallel imaging) method reduces the g‐factor, representing a major improvement on SMS.^85^, ^86^ Blipped‐CAIPI introduces an apparent in‐plane shift between the excited slices, such that a given coil profile is spatially separated in the overlapping slices. In this case, the aliased voxels corresponding to two slices can be more easily separated because the coil profiles appear more distinct (ie, the high‐signal region from an unshifted slice overlaps with the low‐signal region from a shifted slice) (Fig. 8c). SMS‐EPI, in particular blipped‐CAIPI and its variants, has greatly improved the quality of diffusion imaging studies by reducing the tradeoff between spatial and angular resolution. Two high‐profile examples in brain imaging include the Human Connectome Project, where it has enabled a protocol with high spatial and angular resolution at several diffusion weighting “shells”^87^; and the UK Biobank Project, where it has enabled multiple shells with reasonable angular resolution in very limited scan time.^88^ Blipped‐CAIPI SMS‐EPI has also been incorporated with segmented‐EPI^13^, ^17^, ^89^ and reduced FOV (see above). An extension to 3D simultaneously multislab acquisition has also been reported.^90^

Image reconstruction to separate slices in SMS builds strongly on the existing literature in parallel imaging. There are two main categories of reconstruction methods for SMS data: SENSE‐GRAPPA^91^ and Slice‐GRAPPA.^85^ SENSE‐GRAPPA treats the overlapping slices as if they are neighboring in the phase encode direction space over a larger FOV, which casts the slice separation problem in a form that can be solved by conventional parallel imaging reconstructions. With the blipped‐CAIPI scheme, SENSE‐GRAPPA contains artifacts at the concatenation points, which have been avoided by additional zero‐padding^92^, ^93^, ^94^ or concatenating along the readout direction.^95^ By comparison, slice‐GRAPPA trains slice‐specific kernels that project *k*‐space data to one corresponding slice. Slice‐GRAPPA has been shown to be dependent on coil sensitivity rather than the image contrast,^85^ which is a desirable property for diffusion MRI, where the reconstruction is trained on data with no diffusion weighting. Several further refinements to SMS reconstruction have been proposed. The reconstruction kernel in Slice‐GRAPPA has been improved to reduce “leakage” between slices^96^ by training the kernel to block signal from all but one slice. This modification has been crucial for simultaneous slice and in‐plane acceleration, both of which play an important role in data quality. However, the interaction between these two accelerations is still challenging, given that both methods rely on multichannel coils in a similar manner.

RF pulses that excite multiple slices simultaneously are generally referred to as “multiband” (MB) pulses, since they deposit energy at several separate frequency bands. These pulses in general require a higher energy deposition, which is necessarily limited by patient safety considerations. A basic MB pulse is a superposition of multiple conventional RF pulses, leading to an N^2^ increase of the peak RF power for N simultaneously excited slices. This problem is particularly challenging for diffusion MRI due to the use of high‐energy 180° refocusing pulses. RF power can be reduced by optimizing the phases of the superimposed RF pulses^97^ or using a time‐shift scheme^98^; however, these methods only reduce the peak RF power, not the SAR level. The variable‐rate selective excitation (VERSE) algorithm^99^ can be used to improve MB pulses by modifying the gradients to slow down *k*‐space transversal speed during peak power deposition; however, VERSE suffers from RF profile distortion in the presence of strong field inhomogeneity. Another approach is PINS (power independent of number of slices) pulses,^100^ which undersample conventional single‐band pulses in a manner that excites equally separated slices while retaining power deposition comparable to a single‐slice excitation. Drawbacks of PINS pulse include poor slice profiles and limitations on the achievable slice orientation. Improvements include combination of PINS with more conventional MB pulses and methods that improve robustness against $B_{1}^{+}$ inhomogeneity.^101^, ^102^, ^103^ Parallel transmission (pTx) has also been explored for improving SMS acquisition. Using a full pTx‐MB model, a significant reduction on total RF power can be achieved.^104^ Alternatively, a dual‐ring RF array design^105^, ^106^ has been proposed, but suffers from the RF discontinuity between SMS slice stacks.

In summary, SMS enables full‐brain diffusion MRI with acquisition time of 3–7 seconds per volume (dependent on resolution and acceleration factor), enabling high spatial resolution and high angular resolution diffusion acquisition, provided reconstruction challenges such as compatibility with in‐plane acceleration can be addressed. The challenges from RF inhomogeneity and high SAR level are likely to be addressed by novel RF pulse design and pTx technique.

### Fast Diffusion MRI With Compressed Sensing

**Figure 9:**
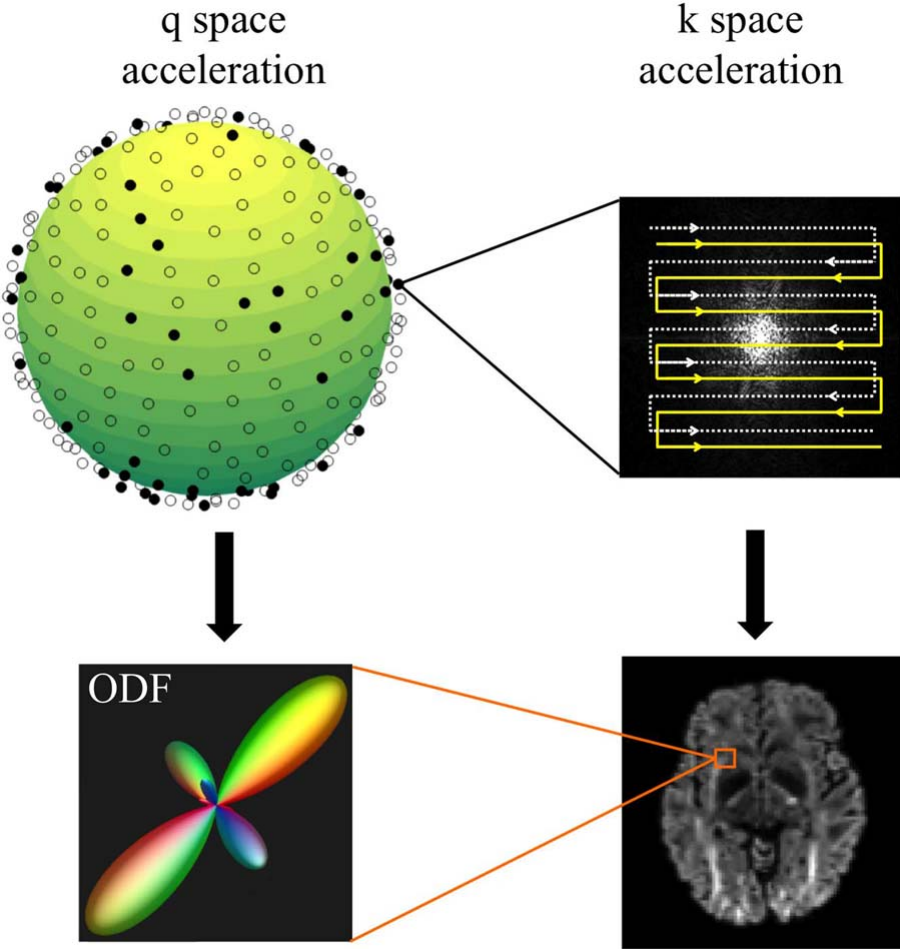
In diffusion MRI exam, a full *k*‐space is acquired at each q‐space sample. The total scan time is determined by the number of q‐space samples and the *k*‐space acquisition time. Therefore, diffusion MRI can be accelerated in both q‐space and *k*‐space, although their objectives are different: in *k*‐space acceleration, it is important to robustly recover the image information from reduced *k*‐space data, whereas in q‐space acceleration, the main target is to achieve accurate fiber orientation estimation (eg, orientation distribution function [ODF]) with fewer q‐space samples.

The total acquisition time of a diffusion MRI exam is determined by the number of diffusion volumes (referred to as “q‐space samples”) and scan time for each volume (*k*‐space). Recently, compressed sensing (CS) methods have been applied to accelerated diffusion acquisition by exploring data sparsity in these two independent domains: q‐space and *k*‐space (Fig. 9).

Density of sampling in q‐space is a key determinant of data quality for diffusion MRI methods that aim to characterize the orientational microstructure of tissue; for example, to enable diffusion tractography (delineation of white matter pathways achieved by following the preferential direction of water diffusion from voxel‐to‐voxel) in the brain. This presents an important challenge for these methods because scan times are in general proportional to the number of q‐space samples acquired (since each direction represents one imaging volume). Recently, CS has been used to improve the reconstruction for acquisitions with a small number of q‐space samples (ie, q‐space undersampling, generally with a pseudorandom sampling pattern). For example, several groups have reported CS methods for reconstruction data acquired with a single b‐value (q‐space “shell”) to capture complex fiber architecture based on a multi‐tensor model^107^ or spherical ridgelets basis.^108^ Other CS methods aim to capture the additional information available from multiple q‐space shells using adaptive dictionaries or a sparsity prior on the diffusion probability density function.^109^, ^110^ These CS methods have reported high‐fidelity recovery of fiber orientation properties from data with undersampling factors ranging from three to eight, where higher accelerations are more readily achievable for simple models (eg, multi‐tensor fits) compared to less constrained models (eg, orientation distribution functions). Note that there are some other developments for CS diffusion MRI,^111^, ^112^, ^113^, ^114^ which are not discussed in the current review due to limited space.

In general, *k*‐space undersampling is only effective for reducing scan time in multishot methods, since 2D single‐shot techniques like SSH‐EPI acquire the entire *k*‐space required for one slice in a very brief time window. To improve the reconstruction fidelity with highly undersampled data, CS reconstructions have been proposed using sparsity priors on diffusion anisotropy,^115^ wavelet representations of the image data,^116^ and Gaussian mixture models of the diffusion process.^117^

Joint k‐q space accelerations, which undersample in both domains, have also been proposed.^117^, ^118^, ^119^ These methods use circulated or randomized *k*‐space undersampling patterns for different q‐space locations to increase the incoherence of signal aliasing, and used CS or low rank models to recover the underlying images. A critical step in the k‐q joint reconstruction is to correct the motion‐induced phase errors, which are inconsistent among q‐space samples. Navigator correction^117^, ^119^ and the MUSE scheme^118^ discussed in previous sections were applied to address this issue.

## Increasing Image SNR

The third major challenge of diffusion MRI that we consider in this review is the intrinsic low SNR, which makes it particularly difficult to achieve high spatial resolution diffusion MRI data. Two dominant methods to enhance SNR of diffusion MRI without significantly increasing the scan time include using imaging sequences with higher SNR per unit time (efficiency) (eg, 3D acquisitions), and the use of ultrahigh field scanner (7T and higher). In this section, the developments of these two approaches for diffusion acquisition are discussed.

### 3D Acquisition

As detailed later on in this section, 3D acquisition methods are attractive because they enable imaging with high SNR efficiency. A further benefit of 3D imaging is the ability to define thin slices using gradient encoding (3D *k*‐space), which produces slices with similar quality of definition to in‐plane voxels, where 2D slices tend to have poor slice definition (eg, warping).

3D diffusion MRI is challenging because *k*‐space encoding in three dimensions can only be accomplished with a multishot approach, meaning that motion‐induced phase errors need to be corrected before combining across shots. In general, phase errors will vary spatially in all directions, implying that a full correction of these errors would require navigation in three dimensions; however, the acquisition of such a 3D navigator would require prohibitive scan time. Several approaches for avoiding the need for navigation in 3D imaging have been proposed. Driven equilibrium returns the magnetization to the longitudinal axis after diffusion preparation in order to enable spoiling of the phase error prior to the 3D readout.^120^ Although driven equilibrium avoids the profound image artifacts associated with phase cancellation, this method still has signal dependence on motion due to corruption of the longitudinal magnetization, which is highly problematic for quantification. Similarly, cardiac gating can reduce the effects of pulsatile motion in 3D imaging,^121^ but residual motion artifacts persist.

Many of the 3D acquisitions for diffusion imaging were first proposed in the context of diffusion weighted SSFP (DW‐SSFP),^122^ which is an intrinsically 3D sequence. Early work explored the use of 1D^123^ and 2D^124^, ^125^ navigation for correcting 3D data. Subsequent work proposed a rotated‐EPI acquisition scheme (TURBINE), which enabled a composite 3D navigator based on cardiac‐synchronized “batching” of 2D readouts.^126^ This approach was extended by including rigid‐body motion correction.^127^ However, a major outstanding challenge faced by DW‐SSFP methods is the disruption of signal formation by motion‐induced phase errors, which results in altered signal magnitude that confounds quantification.

**Figure 10:**
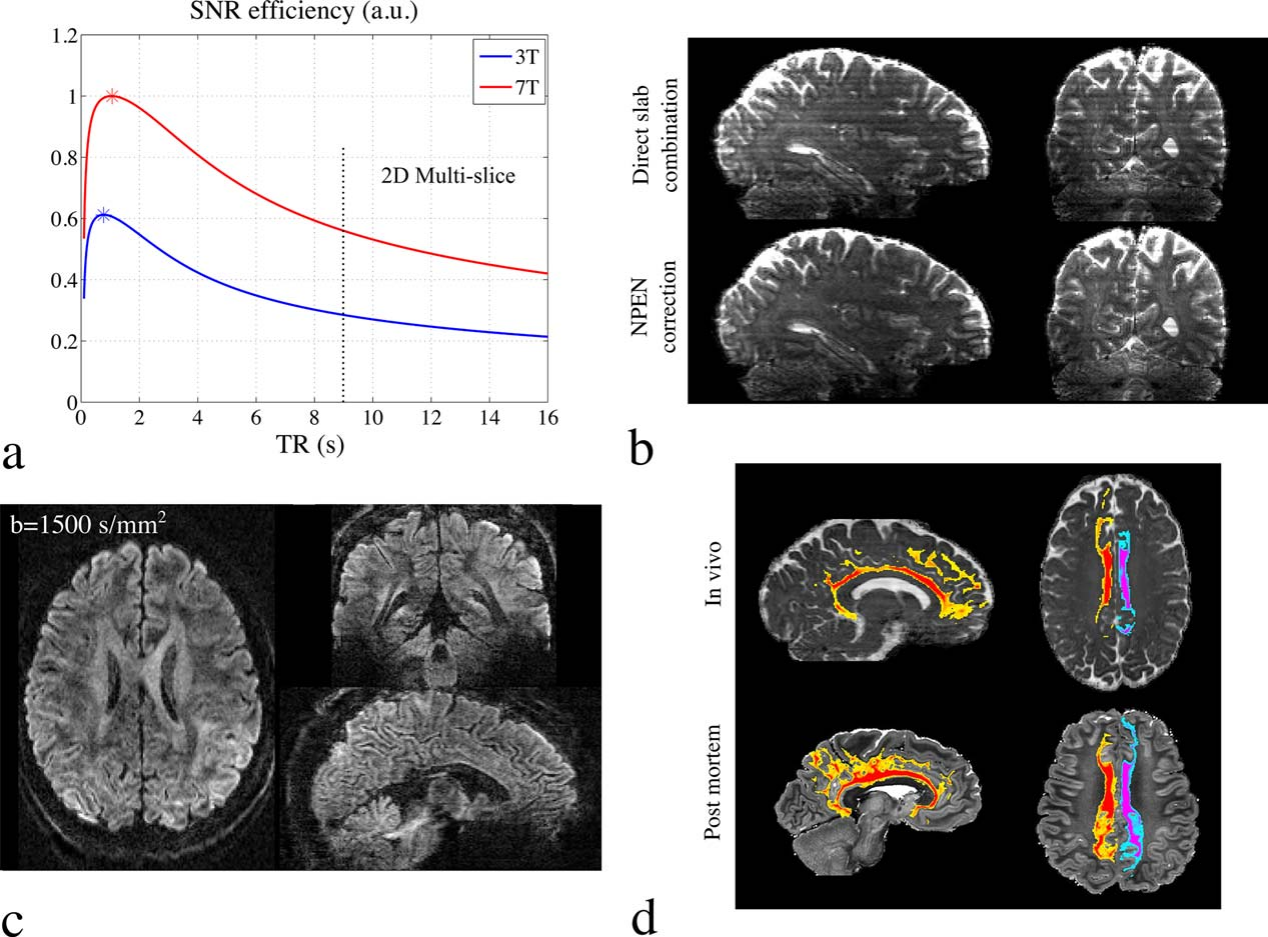
**(a)** SNR efficiency plot for diffusion‐weighted spin‐echo sequence. Here, white matter is analyzed. For the *T*_1_ value of white matter, the optimal TR is between 1–2 seconds, which is not compatible with full brain coverage using conventional 2D multislice acquisitions. Simultaneous multislice acquisition enables a short TR, but still faces limitations at high‐resolution scan. 3D multislab can achieve very short TR (2–3 sec) for full brain coverage, enabling a higher SNR efficiency. **(b)** Slab boundary artifacts in 3D multislab acquisition. First row shows the result of direct slab combination, where all slabs are concatenated with outermost slices discarded. Second row shows the correction using Nonlinear inversion for slab Profile ENcoding (NPEN) method, in which the artifacts are effectively reduced. **(c)** 1 mm isotropic resolution diffusion MRI data acquired at 7T using 3D multislab acquisition, demonstrating high SNR and good anatomical details. **(d)** Tractography of the cingulum bundle from in vivo data (top row) captures essentially the full extent of the cingulum bundle, including temporal and frontal lobe tracts and cortical projections into cingulate cortex. Tractography on postmortem data (from a different study) is shown as gold standard (bottom row).

Recently, development in this area has focused on 3D multislab acquisitions, in which a series of slabs are excited in sequence (similar to 2D multislice imaging but many thick slices), within which slices are defined using gradient encoding (ie, k_z_ encoding as in 3D imaging). As a hybrid 2D/3D method, 3D multislab acquisition can achieve thin slices like 3D imaging while being compatible with the moderate TR (1–2 sec) associated with high SNR efficiency (Fig. 10a).^26^, ^128^ When a thin slab is applied, the motion‐induced phase error along the slice direction can be assumed to be slow varying, thus a 2D navigator can provide sufficiently good correction.^59^, ^128^ The use of a 2D navigator for phase error correction is a key enabler of 3D multislab diffusion acquisition, as it relieves the demanding requirement of 3D navigator acquisition. Several groups have demonstrated the use of 3D multislab acquisitions to achieve high‐resolution diffusion MRI data.^26^, ^41^, ^128^, ^129^, ^130^ A further challenge with 3D multislab acquisition is slab boundary artifacts, which arises because the magnetization experiences variable saturation at the overlap of slab profiles. Several reconstruction methods have been proposed to address this issue, with increasing degrees of sophistication (Fig. 10b).^131^, ^132^, ^133^ A recent study acquiring 3D multislab diffusion MRI data at 7T demonstrated high spatial resolution and high SNR,^130^ as shown in Fig. 10.

### Ultrahigh Field

Because it provides greater signal (polarization of water), ultrahigh field strength enables higher intrinsic SNR, which has great potential for high resolution and/or high b‐value diffusion imaging. However, in order to fully benefit from the increased signal polarization, several challenges must be addressed, including: shorter *T*_2_, which reduces signal level dramatically at the long echo times typical in diffusion MRI; the strong RF transmit field inhomogeneity, which is particularly problematic for refocusing pulses in spin‐echo sequences; increased B_0_ inhomogeneity, which is associated with EPI distortion; and significantly increased SAR level, also associated with refocusing and/or SMS pulses. Despite these challenges, several studies have demonstrated high‐quality diffusion MRI data at 7T.^2^, ^68^, ^130^, ^134^, ^135^ Due to shorter *T*_2_, achieving short echo time is critical for realizing the SNR benefit at ultrahigh field^136^; this has been demonstrated through the combination of parallel imaging and partial Fourier acquisition.^130^, ^135^ The use of rs‐EPI^17^, ^22^ and reduced FOV^66^, ^68^ can further reduce the echo time, as well as reducing EPI distortion. RF transmit field inhomogeneity can be mitigated using dielectric pads,^135^ although this approach inevitably improves some regions while worsening homogeneity in others.^137^ A more comprehensive solution to transmit inhomogeneity would be to use parallel transmission, which can also reduce the SAR level. The combination of 3D multislab acquisition and 7T can further enhance the SNR performance.^130^ In all, diffusion MRI at ultrahigh field is a rapidly advancing research area. Given the intrinsic low‐SNR of diffusion MRI and the increasing desire for higher spatial resolution and b‐value, ultrahigh field is expected to play an important role in future diffusion MRI research.

## Conclusion and Outlook

We have presented an overview of the major technical advances for diffusion MRI acquisition in recent years. Compared to standard 2D SSH‐EPI acquisition, these methods have demonstrated capabilities to enhance data fidelity, reduce acquisition time, and improve SNR. Some of these techniques (rs‐EPI, rFOV, PROPELLER) have already been applied in clinical settings with the aim of minimizing image distortion and blurring for improved diagnostics or disease monitoring in clinically feasible scan times. In contrast, basic neuroscience research applications typically require many diffusion directions with full brain coverage, for which accelerated acquisitions like SMS have already demonstrated major impact. For high isotropic resolution (eg, submillimeter) diffusion scans, 3D acquisition has advantages including the accurate slice profile and SNR efficiency. Finally, diffusion MRI is starting to take advantage of advances in ultrahigh field technology, including improved hardware and RF transmit techniques. While most of these methods have been developed in the brain, diffusion MRI throughout the body has become a major area of research focus, building on these advances for entirely new applications. Further improvement and combination of these techniques should enable more robust diffusion MRI acquisition with higher data quality, providing a powerful tool for new diagnostics, monitoring, and mechanistic studies.
